# Treatment of children and adolescents with hemangioma using propranolol: preliminary results from a retrospective study: Tratamento de crianças e adolescentes com hemangioma com propranolol: resultados preliminares de um estudo retrospectivo

**DOI:** 10.1590/1516-3180.2014.1321575

**Published:** 2014-02-01

**Authors:** Juliana Costa Albuquerque, Rosane Aline Magalhães, Jamille Araújo Félix, Maria Vilani Rodrigues Bastos, Juvenia Bezerra Fontenele, Nádia Mendonça Trompieri, Francisco Helder Cavalcante Felix

**Affiliations:** I Pharmacy Student, Universidade Federal do Ceará (UFC), Fortaleza, Ceará, Brazil; II Employee of the Department of Pharmacology and Physiology, Universidade Federal do Ceará (UFC), Fortaleza, Ceará, Brazil; III PhD. Adjunct Professor, Pharmacy Course, Faculty of Pharmacy, Dentistry and Nursing, Universidade Federal do Ceará (UFC), Fortaleza, Ceará, Brazil; IV MD, MSc. Pediatrician in Walter Cantídio University Hospital, Pediatrician and Hemato-oncologist in the Pediatric Cancer Center, Albert Sabin Children's Hospital, Fortaleza, Ceará, Brazil; V MD, MSc. Pediatrician and Hemato-oncologist in Albert Sabin Children's Hospital, Fortaleza, Ceará, Brazil

**Keywords:** Hemangioma, capillary, Propranolol. Retrospective studies, Treatment outcome, Medical oncology, Hemangioma capilar, Propranolol, Estudos retrospectivos, Resultado de tratamento, Oncologia

## Abstract

**CONTEXT AND OBJECTIVE:**

Hemangiomas are the commonest vascular tumors during childhood. In 2008, the effect of propranolol for treating capillary hemangiomas was demonstrated. Other similar results followed, showing that it rapidly reduces lesion volume. The objective here was to evaluate children and adolescents with hemangiomas that were treated with propranolol.

**DESIGN AND SETTING:**

Retrospective study, conducted in a children's hospital.

**METHODS:**

: Patients aged 0-19 years with or without previous treatment, who were treated between January 2009 and December 2010, were included. The response was assessed by comparing the lesion appearance between the start of treatment and the last consultation. We considered partial or complete responses as the response to treatment.

**RESULTS:**

: Sixty-nine patients with a median follow-up of 11 months (mean age: 31 months) were included. Of these, 58 patients were recently diagnosed and 11 had had previous treatment. A response (partial or complete) was seen in 60 patients (87%). Among the capillary hemangioma cases, responses were seen in 50 out of 53 (94%), while in other lesion types, it was 10 out of 16 (63%) (P = 0.3; chi-square). Responses in patients less than one year of age were seen in 37 out of 38 (97%), whereas in those over one year of age, in 23 out of 31 (74%) (P = 0.4; chi-square). Side effects were uncommon and mild.

**CONCLUSIONS:**

Propranolol seemed to be effective for treatment of hemangiomas in children and adolescents, and not just in the proliferative stage, with responses in almost all the patients.

## Abstract (pt)

**CONTEXTO E OBJETIVO:**

Hemangiomas são os tumores vasculares mais comuns da infância. Em 2008, foi demonstrado o efeito do propranolol no tratamento de hemangiomas capilares. Outros relatos similares seguiram-se, demonstrando seu rápido efeito na redução do volume das lesões. O objetivo foi avaliar crianças e adolescentes com hemangioma tratadas com propranolol.

**TIPO DE ESTUDO E LOCAL:**

Estudo retrospectivo, conduzido em hospital infantil.

**MÉTODOS:**

Foram incluídos pacientes entre 0-19 anos, com ou sem tratamento prévio, tratados entre janeiro de 2009 e dezembro de 2010. A resposta foi avaliada comparando-se o aspecto da lesão entre o início do tratamento e a última consulta. Consideramos resposta parcial ou completa como resposta ao tratamento.

**RESULTADOS:**

Sessenta e nove pacientes foram incluídos, com uma mediana de acompanhamento de 11 meses (idade média: 31 meses). Destes, 58 pacientes eram recém-diagnosticados e 11 tinham tratamento prévio. Resposta (parcial ou completa) foi verificada em 60 pacientes (87%). Entre os hemangiomas capilares, a resposta foi de 50 em 53 (94%), enquanto em outros tipos de lesões, a resposta foi de 10 em 16 (63%) (P = 0,3; teste de qui-quadrado). A resposta em pacientes com até 1 ano de idade foi de 37 em 38 (97%), e naqueles com mais de 1 ano foi de 23 em 31 (74%) (P = 0,4; qui-quadrado). Efeitos colaterais foram incomuns e leves.

**CONCLUSÕES:**

Propranolol parece ser efetivo no tratamento de hemangiomas em crianças e adolescentes, não apenas na fase proliferativa, com resposta em quase todos os pacientes.

## INTRODUCTION

Hemangiomas are formed by proliferation of blood vessels and are the commonest vascular tumors during childhood, affecting approximately 3-10% of Caucasian children.^1^ They occur more frequent in females (1:1.4-3.0), and in white non-Hispanic children. Their causes are unknown, with the exception of rare genetic syndromes, in which hemangioma is frequent.^2^ Histologically, hemangiomas are a heterogeneous group,^3^ although the most common type is known simply as infantile or capillary hemangioma. 

Hemangiomas display different growth phases over the course of their evolution. Initially, there is a rapidly proliferating phase lasting for up to six months. During this phase, the lesion becomes more erythematous and violacious. Larger, deep hemangiomas may proliferate up to the age of two years. A stationary phase ensues, during which the hemangioma grows in proportion to the child. This is followed by an involutive phase that lasts for up to five years in most cases. Involutive hemangiomas change color to gray. Maximum involution occurs in approximately 50% of children by the age of five years and in 90% of children by the age of nine. Some 20% to 40% of patients keep residual changes of the skin, such as laxity, discoloration, telangiectasia, fibrofatty masses or scarring.^4^

Most hemangiomas are small and never complicated, and they are adequately managed through clinical observation alone. A number of pharmacological interventions have been used for the approximately 10-30% of these patients who present complications. The majority of these treatments have never been properly clinically evaluated, aside from reports on small numbers of cases. The first-line therapy has been oral corticosteroids for many years. Other treatments include topical or intralesional corticosteroids, interferon alpha and vincristine. In the absence of any randomized controlled clinical trials, the dosing, time duration and efficacy of oral corticosteroids are determined empirically at best.^3^ ^,^ ^4^

In 2008, a letter published in the New England Journal of Medicine first reported use of propranolol for treating childhood hemangiomas.^5^ After this initial information, other reports on cases successfully treated with propranolol were published, and the initial article has been cited around 140 times (Google Scholar survey in January 2011). Since January 2009, we have been treating pediatric patients with hemangiomas using off-label oral propranolol in our institution.

## OBJECTIVES

To describe the therapeutic effects of propranolol, in a cohort of children and adolescents with hemangioma from a single institution. 

## METHODS

We planned to evaluate the response of children with hemangiomas to treatment with propranolol. Adverse events reported during the treatment would be recorded. The research project was approved by our institution's Ethics Review Board in 2009. The project is still at the data-gathering phase. This report presents partial preliminary data, according to our database in June 2011.

Parents or guardians received detailed explanations about the treatment and this was started after informed consent had been obtained. A retrospective analysis of the medical records was undertaken, using a semi-structured questionnaire. We included patients ranging in age from 0 to 19 years with a diagnosis of hemangioma, with or without prior treatment. We excluded patients with asthma (as informed by parents).

We started administering treatment with propranolol between January 2009 and December 2010. The response was assessed by comparing the status at the start of treatment and at the last appointment, measuring the two largest diameters of the lesions. Patients with unmeasurable lesions were evaluated by means of a qualitative assessment made by one of the attending physicians (i.e. one of the present authors). The response was classified as stable disease (< 25% variation), partial response (25-95% reduction) or complete response (> 95% reduction). We considered both partial and complete responses to be responses to treatment. Objective measurements were made by means of direct measurement, ultrasound imaging, computed tomography (CT) or magnetic resonance imaging (MRI), depending on the accessibility of the lesion. Deep lesions not measurable by means of ultrasound imaging were followed by means of serial CT or MRI (the number of CT scans was kept to the minimum necessary for response assessment, and was typically two to three scans). The chi-square test was used to compare responses between different groups of patients (infantile hemangiomas versus other types and children less than one year of age versus children older than one year).

## RESULTS

We included 69 patients with a mean follow-up of 11 months. The average age at the time of starting the patients' first treatment was 31 months, ranging from one month to 19 years. The median was 8 months. The average age at the time of starting the treatment, for patients with residual lesions or those refractory to prior therapy, was three years, ranging from two months to 16 years. A total of 38 patients started treatment at the age of less than one year, while 31 commenced after completing one year of age. The dose used was 0.5 to 4.0 mg/kg per day, starting with 0.5 mg/kg for all patients in the first week, with weekly increases so as to reach up to 2.0 mg/kg per day. For patients with no initial response over the first 2-3 months, the dose was increased to 4.0 mg/kg per day. The dosing interval was 8 or 12 h. 

**Figure 1:**
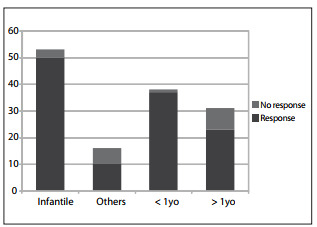
Treatment response in patients with infantile hemangiomas, other types of vascular lesions, patients less than one year of age or over one year of age (number of patients).

Fifty-eight patients had not been treated previously, while 11 presented residual lesions or had been refractory to previous therapy. A response (partial or complete) was seen in 60 patients (87%). Forty-six patients were female and 23 were male (ratio 1:2). The lesions were classified as infantile hemangioma (53), cavernoma (three), syndromic hemangioma (four), congenital lesions or others (nine). Responses were seen in 50 out of 53 cases of infantile hemangioma (94%) and in 10 to 16 cases of the other hemangioma types (63%) (P = 0.3; chi-square). Responses were seen in 37 out of 38 patients who started treatment before reaching the age of one year (97%), whereas the proportion was 23 out of 31 patients who started treatment after reaching one year of age (74%) (P = 0.4; chi-square) (Figure 1). 

Side effects were uncommon and mild, and the treatment was not discontinued for any child in the series because of side effects. The dose was reduced in some cases, due to side effects. Sixteen patients reported possible side effects. The most common were transitory dyspnea (four patients), cold extremities (two patients), precordial pain (two patients) and slow weight gain (two patients). One patient presented symptomatic hypoglycemia (56 mg/dl) after prolonged fasting. This patient did not have any response to the treatment and it was withdrawn. 

**Figure 2:**
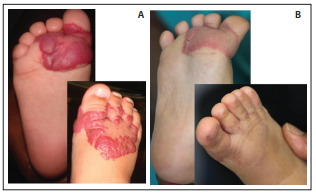
Infantile hemangioma on the feet of a threemonth old child A. before treatment, and B. after one year of treatment. Complete remission is apparent. Residual telangiectasia remained.

Figure 2 illustrates a typical case of unmeasurable infantile hemangioma with complete response to treatment. 

**Figure 3:**
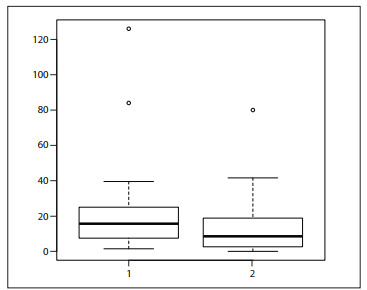
Boxplot of lesion sizes (surface area estimates) at first measurement (1) and last measurement (2). The graph shows the median (band), 25th and 75th quantiles (bottom and top of box), sample minimum and maximum (whiskers) and outliers (dots).

**Figure 4:**
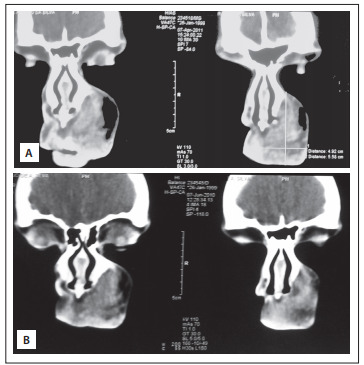
Computed tomography scans on a patient with a congenital facial lesion, contrast enhancing, non-involutive. Imaging and clinical history were used for diagnosing non-involutive congenital hemangioma (NICH). Propranolol treatment did not change the estimated lesion size. Upper panel (A) shows lesion after the treatment, whereas lower panel (B) shows the lesion 10 months earlier.

Table 1 shows information about the measurable lesions. Figure 3 shows the change in lesion measurements, from the first to the last objective evaluation. Figure 4 illustrates the CT scans of a case of non-involutive congenital hemangioma (NICH) that remained stable over the course of the treatment time (i.e. no response).

**Table 1:**
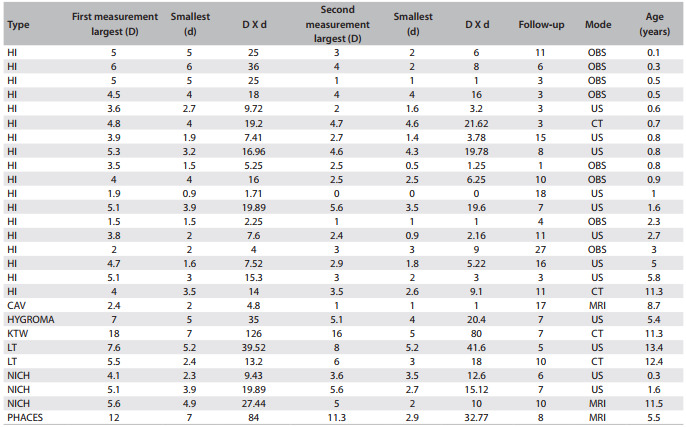
Lesion size in the subgroup of patients with measurable disease

Types of lesions: capillary hemangioma (HI), cavernous hemangioma (CAV), lymphangioma (HYGROMA), Klippel-Trenaunay-Weber syndrome (KTW), vascular lesions identified in older children (probably malformations - LT), non-involutive congenital hemangioma (NICH), PHACES syndrome (PHACES). First and last measurement are shown, each depicting largest diameter (D), smallest diameter (in fact, the largest perpendicular diameter, d) and the product of the two diameters (estimate of surface area). Follow-up time: from beginning of treatment until last measurement. Mode of examination: direct observation and measurement (OBS), sonography (US), computed tomography (CT), magnetic resonance imaging (MRI)

## DISCUSSION

In this preliminary retrospective study, treatment with propranolol was related with a response (consisting of lesion reduction) in most of the children with hemangiomas. It seemed that there was a greater chance of a response in children with infantile hemangiomas who were less than one year of age, in contrast to patients with other types of hemangiomas or who were over one year of age. However, this difference was not statistically significant. The statistical power of the comparison of numbers of responders between those aged less than and more than one year was 80% (data not shown). This indicates that the chance of type II error was small and that there was probably no real difference in the number of responders between children aged less than and more than one year. However, this evaluation did not differentiate between a partial response (defined less strictly in the present study, such that responses that are regarded as minor were also included) and a complete response. One of our goals is to complete the data-gathering for the entire cohort analysis with detailing of the two different outcomes (partial or complete). However, our preliminary results already show that patients who have outgrown the so-called "proliferative phase" of hemangioma development still have a potential for response that should not be underestimated.

The small number of patients with lesions other than infantile hemangiomas does not allow any conclusion about their response potential. However, the statistical power of this comparison was 83%. This heterogeneous group of patients included three patients with cavernous hemangiomas (histologically determined), four syndromic patients with apparently typical infantile hemangiomas (PHACES and Klippel-Trenaunay-Weber syndromes) and nine patients with congenital hemangiomas or late-onset lesions. It is possible that patients with other forms of vascular tumors closely related to infantile hemangiomas may also have a potential for response. It remains to be determined whether this response potential is actually lower than that of patients with infantile hemangiomas.

Infantile hemangiomas have typical presentation and evolution.^2^ They express a homogeneous group of immunohistochemical markers, including GLUT1 (glucose transporter 1), which is a surface protein expressed by erythrocytes and the endothelium of infantile hemangiomas.^6^ It is possible that propranolol has a specific effect on lesions that express GLUT1, regardless of its presentation and stage of development. Indeed, infantile hemangiomas have been found to express GLUT1 in both the proliferative and regressive phases.^6^ In contrast, non-evolutive congenital hemangiomas constitute a group of lesions that are clinically and histologically distinct and do not express this marker.^7^ In our series, the patients with congenital hemangiomas (clinical and radiological diagnoses) showed little or no response, unlike most other patients (data not shown). Perhaps the lesions with different presentations or evolution that respond to propranolol are actually GLUT1 positive (+) hemangiomas.

The mechanism of action of propranolol in infantile hemangiomas is still the subject of speculation. Initially, the idea was that this effect could be mediated by binding to beta-adrenergic receptors, leading to reducing of pro-angiogenic factors like VEGF (vascular endothelial growth factor) and b-FGF (fibroblast growth factor beta).^8^It has already been shown that infantile hemangiomas express adrenergic receptors and are closely related to sympathetic innervation.^9^ There has been speculation that, in particular, inhibition of beta-2 adrenergic receptors may lead to vasoconstriction, anti-angiogenesis (via inhibition of VEGF) and induction of apoptosis in hemangiomas.^10^ However, no experimental evidence has corroborated these hypotheses. Other possible molecular pathways that are involved in vascular tonus and endothelial proliferation and which could directly or indirectly function as targets of propranolol include: cAMP/PKA, leading to increased VEGF/b-FGF;^11^ inhibition of vasodilation through reducing the release of NO mediated by beta-3 receptor ligands;^12^ and VEGF production regulated by NF-kB, which relates to the effect of steroids on hemangiomas.^13^ Recently, involvement of elements of the renin-angiotensin-aldosterone system has been suggested, via inhibition of the renal renin-angiotensin-aldosterone system by propranolol, thus leading to inhibition of proliferation of endothelial progenitor cells that express receptors for VEGF and the CD34 marker.^14^ However, an anecdotal reference to alleged direct binding of propranolol with GLUT1 has no scientific basis.^15^

Regardless of the mechanism of action, it is now indisputable that propranolol has an important effect on infantile hemangiomas, such that it causes their rapid regression.^16^ Other groups have also reported similar results,^17^ ^,^ ^18^ in which they showed that the treatment rapidly induced stabilization of lesion proliferation and reduction of the volume of lesions in 100% of the patients. A review of several worldwide series^19^ of between one and 58 patients reported that propranolol had been effective in most cases. In 205 pooled cases, there was an "excellent response" in 42 cases, while 69 were classified as "good" or "moderate" or "partial response", 56 had responses that were not quantified and 10 did not respond at all or "deteriorated" or showed "mild recurrence". The rest of the patients' responses were not described. This corresponds to an 82% response rate and a 5% refractoriness or relapse rate. The response in individual series has ranged from 47 to 100%. Doses and administration schedules have varied little: 1-3 mg/kg per day, either with a gradual increase or starting at full dose. The duration of treatment reported has varied considerably, from two to 18 months, which may explain some of the variability of the results. A double-blind, randomized clinical trial of propranolol reported that the treatment was effective in 90% of the 19 children (four months to five years of age), who were treated with 2 mg/kg per day at 8 h intervals. The treatment on the lesions caused them to soften and change color from red to purple within 24 h, stopped their growth in 2-30 days and caused a rapid volume reduction by 4-8 weeks. Thereafter, the reduction of the residual lesions was slower. The trial showed that there was a statistically significant reduction of redness and elevation of infantile hemangiomas.^20^ There is no recommended length of treatment, but it has been shown^20^ that treating for a minimum of six months and at least until one year of age may prevent recurrences.

These data are comparable with the results from our series. We observed responses in 87% of our 69 patients. Most of the published series included only infants with capillary hemangiomas.^19^ The original series included patients aged up to four years, and the only randomized trial completed so far included children of up to five years of age.^17^ ^,^ ^20^ In our series, most of the patients were under one year of age, and 75% were 5 years of age or less, and thus our results are directly comparable with the previously published series. A small number of older children and adolescents (most with lesions other than capillary hemangiomas or residual/refractory hemangiomas) were included in this series. To our knowledge, this is the first report to include patients over five years of age treated with propranolol. Surprisingly, some patients in the older subset did respond to the treatment, thus indicating that propranolol may have some therapeutic utility in these patients.

β-blockers have a well-documented safety and side-effect profile. Over the course of 40 years of clinical use at therapeutic doses in children less than seven years of age, there have been no cases of mortality and no serious cardiovascular events.^20^ The safety of propranolol was not addressed in our retrospective series. However, we recorded few side effects, and none requiring treatment discontinuation. Previously published series reported variable incidence of adverse events, ranging from none recorded^20^ to two thirds of the patients (a Chinese series that reported diarrhea possibly caused by the oral formulation).^19^ Adequate phase I studies on propranolol use among children with hemangiomas may be warranted, in order to ascertain the real adverse event frequency in these patients.

Our report is, as far as we are aware, the largest single-center series so far published in the literature and is one of the few that included syndromic patients with hemangiomas or lesions other from infantile hemangiomas. We were able to reproduce the good results reported by other groups, although we showed that a small number of patients were refractory. These results may have implications for treatments for infantile hemangiomas, given that current practice is to use steroids as first-line therapy in cases of complicated hemangiomas.

Regarding syndromic patients or other types of hemangiomas, and older patients, it is still too early to say for sure whether patients within this heterogeneous group can also benefit from therapy with propranolol. Moreover, it can be hypothesized that propranolol acts specifically on GLUT1-positive lesions, regardless of their clinical presentation. These are interesting directions for future basic and clinical research.

## CONCLUSIONS

Propranolol seemed to be effective in treating hemangiomas in children of all ages, and not only in the proliferative stage of the lesions (up to one year of age), with a high response rate. The outcome varied with the type of lesion, and with the age (difference not statistically significant). Infantile hemangiomas in infants under one year of age showed responses in nearly all patients. Randomized clinical trials are necessary to confirm this finding and to assess the safety of the intervention as well.
